# The use of haplotype-specific transcripts improves sample annotation consistency

**DOI:** 10.1186/2050-7771-2-17

**Published:** 2014-09-30

**Authors:** Nicole Hartmann, Evert Luesink, Edward Khokhlovich, Joseph D Szustakowski, Lukas Baeriswyl, Joshua Peterson, Andreas Scherer, Nirmala R Nanguneri, Frank Staedtler

**Affiliations:** 1 Novartis Institutes for BioMedical Research (NIBR), Biomarker Development, Fabrikstrasse 10.13, CH-4002 Basel, Switzerland; 2 Spheromics, Kontiolahti, Finland

**Keywords:** HLA-DQA1, HLA-DRB4, Microarray quality control (QC), Biomarkers, Dip test, mRNA

## Abstract

**Background:**

Exact sample annotation in expression microarray datasets is essential for any type of pharmacogenomics research.

**Results:**

Candidate markers were explored through the application of Hartigans’ dip test statistics to a publically available human whole genome microarray dataset. The marker performance was tested on 188 serial samples from 53 donors and of variable tissue origin from five public microarray datasets. A qualified transcript marker panel consisting of three probe sets for human leukocyte antigens HLA-DQA1 (2 probe sets) and HLA-DRB4 identified sample donor identifier inconsistencies in six of the 188 test samples. About 3% of the test samples require root-cause analysis due to unresolvable inaccuracies.

**Conclusions:**

The transcript marker panel consisting of HLA-DQA1 and HLA-DRB4 represents a robust, tissue-independent composite marker to assist control donor annotation concordance at the transcript level. Allele-selectivity of HLA genes renders them good candidates for “fingerprinting” with donor specific expression pattern.

## Background

Clinical molecular research and biomarker development rely on a high level of data quality. Ensuring data quality extends beyond the establishment of reproducible technical processes involved in measurement of variables. Obtaining accurate clinical metadata is of utmost importance for meaningful clinical research, as they are necessary for finding clinical disease-treatment or disease-biomarkers relationships [1]. Drawing conclusions based on incorrect metadata can have detrimental consequences in short-term or long-term patient care. Typical sample annotation errors may be due to sample mix-ups, database entry errors, or subjectivity, e.g. grading of a biopsy. In pharmacogenomics analyses, unrecognized annotation errors or sample mix-ups impact any supervised statistical analysis, such as certain steps during biomarker discovery and qualification, and patient stratification. Estimates of sample mix-ups or annotation errors in clinical datasets range up to 18% [2]. Several statistical approaches have been devised to address annotation uncertainty during classifier development [3-5]. These sophisticated and extremely valuable approaches are applied as part of a later phase the analytical process. To begin addressing annotation uncertainty already at the database level, we have recently reported on a transcript marker for gender annotation which can be applied to clinical datasets of whole genome microarrays immediately after the generation of data [6]. The so-called “REDKX” gender marker is based on heterosome genes with gender-dependent expression-characteristic. Application of this marker informs about the correctness of the gender annotation of a donor. In addition, control for inter-individual sample mix-up clinical datasets with multiple, e.g. longitudinal, sampling is an absolute must, and gender annotation QC is not sufficient to that goal, since sample mix-ups between individuals of the same gender would go undetected.

Of particular interest are genes with largely unchanged expression levels in samples of a donor, which would have a significantly different expression level in another donor. At best, those probe sets would display an inter-individual bimodal “on/off”-expression characteristic. Bimodal distribution of (signal intensity) data is a deviation from the assumed normal distribution of measurements within a population, and can be recognized by the presence of two modes, each characterized by a peak. Bimodality can occur by differential expression, heterosome-specific expression, or, as observed e.g. in cancer biology, by genomic lesions in some donors but not in others [7]. Candidate genes with bimodal expression characteristics exhibit heterosome-specific and/or haplotype-specific expression. Some Affymetrix probe sets were designed in polymorphic mRNA sequences and are (unintentionally) haplotype specific. Messenger RNA (mRNA) derived from a donor with a certain haplotype or polymorphism does not hybridize with the same affinity to a locus of different haplotype or lacking the polymorphism, resulting in an extremely different expression level for the latter sample. The reason for the decreased or increased affinity is of technical nature: hybridization of a labelled mRNA fragment to a site with a single nucleotide polymorphism (SNP) causes nucleotide mispairing, leading to the creation of a virtual bubble at the site of the SNP. The result is a less stringent binding of the mRNA fragment to the probe target, and lower signal intensity. Hence, sites with SNPs could be used for indirect genotyping, or “fingerprinting” [8,9].

In order to systematically assess deviation from unimodality of individual probe sets we have utilized the principle of the dip statistic. The dip test had been proposed as a test statistic for unimodality [10]. It estimates the maximum difference between the empirical distribution function and the unimodal distribution function that minimizes that maximum difference. In order to assess the significance of the Dip Test statistic, we ran 1 × 10^6^ simulations, where a sample of the same size as the dataset in question, was drawn from a normal distribution, and a Dip Test applied to each of the draws in order to generate 1 × 10^6^ simulated Dip Test statistics. As a result, we were able to derive an empirical p-value for each probe set. Here we report on the application of the Hartigans’ dip test to transcriptome wide clinical gene expression microarray datasets, in the search of probe sets which would help flag samples with potential donor-ID annotation mix-up.

## Results

**Figure 1:**
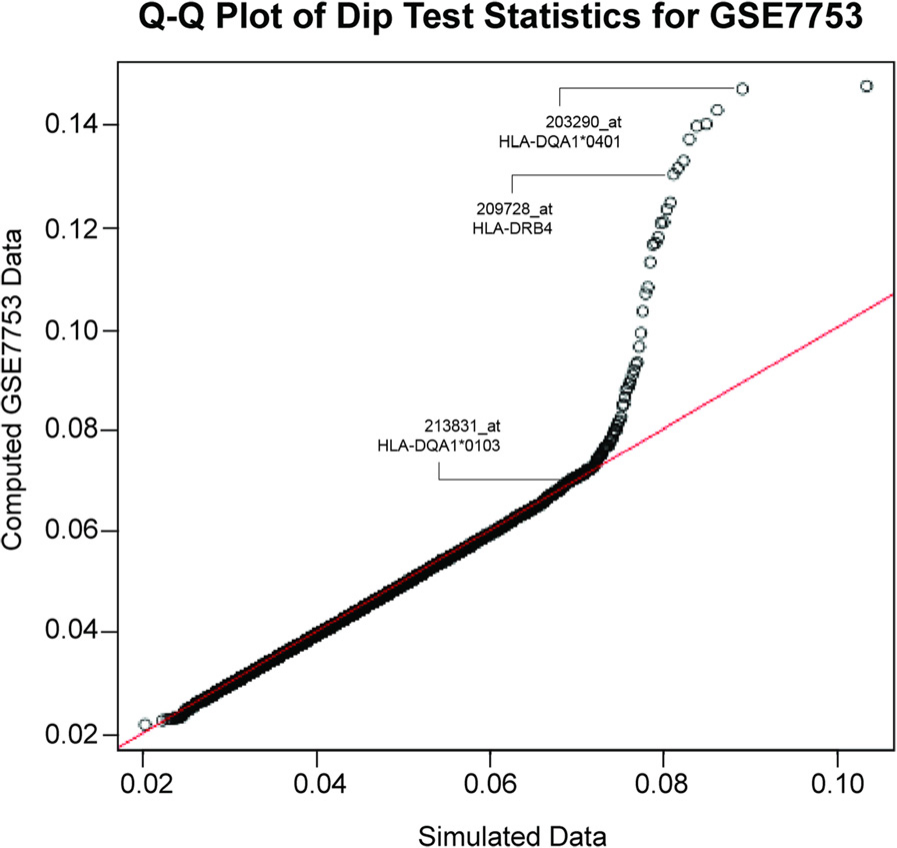
**Quantile-quantile plot of Hartigans’ dip test statistics.** The line of identity (in red) indicates unimodal distribution of data. Simulated data are distributed along this line, while some of the probe sets from the dataset GSE7753 deviate from unimodal distribution. Three candidate marker probe sets, 203290_at (HLA-DQA1*0401), 213831_at (HLA-DQA1*0103), and 209728_at (HLA-DRB4) are pointed out.

**Figure 2:**
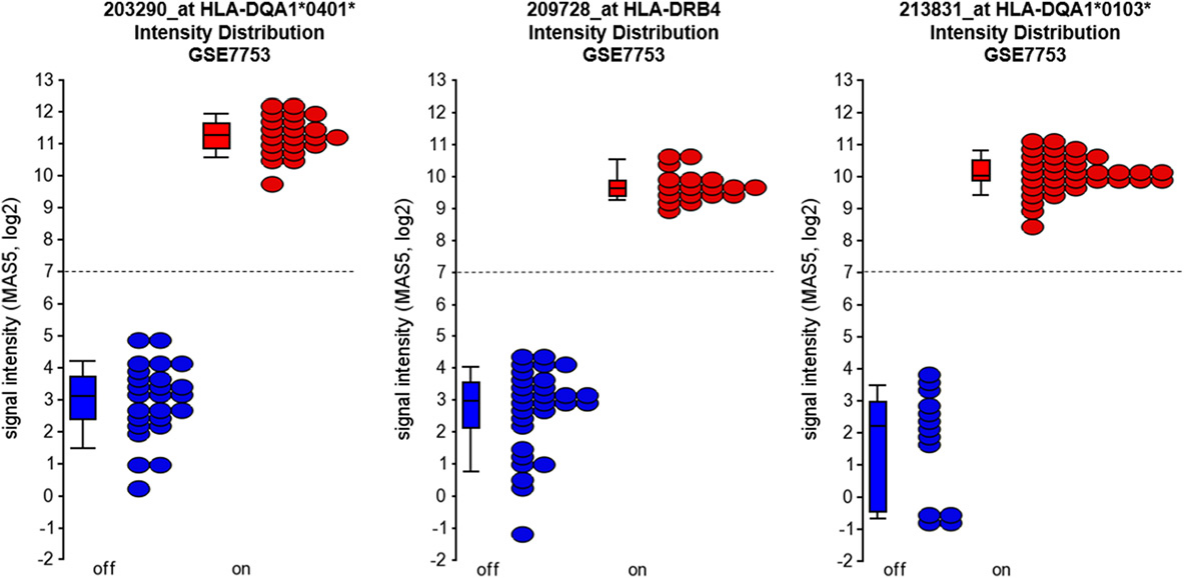
**Bimodal intensity distribution of three candidate marker probe sets in the dataset GSE7753.** The training set consisted of 47 samples. Horizontal lines on the Y-axes indicate the intensity thresholds which were empirically determined for each probe set separately. The box plots represent (from bottom to top) the 10th, 25th, 50th, 75th and 90th percentile of the distribution. Number of bins = 50.

The transcript marker was trained on a publically available dataset of 47 Affymetrix HG-U133_Plus_2 microarrays from a study of systemic juvenile idiopathic arthritis (GSE7753, http://www.ncbi.nlm.nih.gov/geo) [11]. After normalization and intensity filtering (see Methods), 21,044 probe sets entered the Hartigans’ dip test. To compute empirical p-values assessing the significance of an individual dip test statistic value, the dip test statistic was computed for a simulated dataset of 47 samples, the same sample number as the GSE7753 dataset, and 1 × 10^6^ permutations per probe set. Empirically we filtered those probe sets with a p-value < 0.001. Another selection criterion was the minor allele frequency. The selected marker should have a minor allele frequency of less than 50%. Probe sets with the smallest p-values are listed in Table 1. The list is populated with many bimodally expressed genes located on one of the heterosomes, such as RPS4Y1, EIF1AY, DDX3Y, KDM5D, and XIST (the genes of the REDKX gender QC marker [6]), but also probe sets for genes located on autosomes, such as human leukocyte antigen (HLA)-genes HLA-DRB4 (209728_at), and HLA-DQA1 (203290_at). Further investigation revealed that the nucleotide sequence of probe set 203290_at aligns to a highly polymorphic region of the 3’ untranslated region of HLA-DQA1, and is identical to the DQA1*0401 allele, but contains at least one probe with SNPs present in alleles *0101, *0102, *0103, *0201, *0301, and *0501 (Table 2, left panel). The presence of polymorphic SNP sites in a probe set target region is a feature which may confer “fingerprinting”-quality. Inspection of the dip test results lead to the identification of another probe set for HLA-DQA1, 213831_at (p-value 0.006). The 213831_at probe set was further pursued, revealing sequence identity to DQA1-allele *0103 and single nucleotide polymorphisms in at least one probe for the other alleles mentioned above (Table 2, right panel). Thus, both HLA-DQA1 probe sets 203290_at and 213831_at met an important criterion of candidate fingerprinting genes, allele-selectivity. The candidate marker panel was composed of three probe sets: 203290_at (HLA-DQA1*0401 allele), 209728_at (HLA-DRB4), 213831_at (HLA-DQA1*0103 allele). The dip test data for the candidate marker probe sets are shown in Figure 1. The simulated dip data were randomly distributed, as they fall onto the identity line in the quantile-quantile plot (Q-Q plot). The computed dip test data for GSE7753 largely followed the random distribution, but some probe sets including the candidate markers deviate from the unimodal distribution. Figure 2 illustrates the bimodal signal intensity distribution of the three probe sets in the dataset GSE7753, and indicates the cut-off value of 7 (log2 scale) which has been developed empirically after visual inspection of the data. The measured signal intensity per probe set is then flagged with a 1 if it surpasses the threshold or 0 if it does not. Samples from the same donor are expected to have the same score. Since the same score may apply to different donors, one has to assume a certain leakiness of this scoring system, as the same score may apply to different donors. To increase the power to detect donor annotation inconsistencies, we strongly recommend using the REDKX marker in addition to the candidate HLA-score proposed in the present study. We have tested the HLA-score along with the REDKX gender QC marker in five publically available datasets of 188 samples from 53 donors. The normalized data for all samples can be accessed in Additional file 1: Table S1. Table 3 exemplifies many aspects of the performance of the donor ID-marker: (1) differentiation between donors, (2) identification of mislabelled samples, (3) providing additional information, where the REDKX QC marker is insufficient for donor discrimination, and (4) support of the REDKX gender QC. (1) The HLA-score for all four samples of donor 55 are the same, no quality flags for either the HLA-score or the REDKX gender score. Also, the HLA-score of the four samples from donor 54 is the same, but different from the score of donor 55. The result for those 8 samples indicates a high probability that those samples indeed come from different donors. (2) For donor 45, the HLA-score for time point 1 is different from the other HLA-score of the other three samples assigned to this donor, because the intensity value of 187 for 209728_at in the time point 1 sample is well above the threshold of 128, yielding a value of 1 in the HLA-score. The intensity values in the other samples are below the threshold, and yield a 0. This HLA-score difference should prompt the user to investigate and look closer at the data. In our experience, a slight transgression of the threshold is not critical, considering that all other marker intensity values follow a similar pattern as in the case of donor 45. (3) Two incidences were found in two samples from donor 35. Strikingly, three different HLA-marker scores are found for the four samples of this donor. The sample time point 1 is interesting, as not only the HLA-score is different from the rest of the HLA-scores for this donor, but also the REDKX gender marker indicates that this sample belongs to a female, not to a male, as the other three samples. The sample of time point 4, which comes from a male as shown by the REDKX marker, is again very different from the two other samples from (a) male(s), attributed this donor. The intensities for two probe sets of time point 4 of donor 35 are about 10 to 46 times different from the corresponding values in time point 2 and 3. This intensity pattern should raise a flag and initiate a follow-up investigation to determine whether this sample is indeed what the annotation claims. (4) Both the REDKX panel as well as the HLA-score indicate that the sample from time point 2 of donor 32 may be from a different individual. The flag would initiate follow-up investigations of the cause.

**Table 1:** Result of Hartigans’ dip test

**Probeset ID**	**Gene symbol**	**Entrez gene ID**	**Cytoband**	**Dip statistic**	**Empirical p-value**
*205000_at*	*DDX3Y*	8653	Yq11	0.147	<1.00E-06
**203290_at**	**HLA-DQA1**	3117	6p21.3	0.147	<1.00E-06
228492_at	USP9Y	8287	Yq11.2	0.143	<1.00E-06
232618_at	TXLNG2P	246126	Yq11.222	0.140	<1.00E-06
*201909_at*	*RPS4Y1*	6192	Yp11.3	0.139	<1.00E-06
**209728_at**	**HLA-DRB4**	3126	6p21.3	0.137	<1.00E-06
223646_s_at	TXLNG2P	246126	Yq11.222	0.133	<1.00E-06
*224588_at*	*XIST*	7503	Xq13.2	0.131	<1.00E-06
*206700_s_at*	*KDM5D*	8284	Yq11	0.130	<1.00E-06
*204409_s_at*	*EIF1AY*	9086	Yq11.223	0.124	<1.00E-06
*205001_s_at*	*DDX3Y*	8653	Yq11	0.123	<1.00E-06
*214218_s_at*	*XIST*	7503	Xq13.2	0.121	<1.00E-06
*227671_at*	*XIST*	7503	Xq13.2	0.120	<1.00E-06
*224590_at*	*XIST*	7503	Xq13.2	0.118	<1.00E-06
231592_at	TSIX	9383	Xq13.2	0.117	<1.00E-06
*221728_x_at*	*XIST*	7503	Xq13.2	0.116	<1.00E-06
211149_at	UTY	7404	Yq11	0.113	<1.00E-06
226736_at	CHURC1	91612	14q23.3	0.108	<1.00E-06
235446_at	---	---	---	0.107	<1.00E-06
1560263_at	---	---	---	0.103	2.00E-06
223645_s_at	TXLNG2P	246126	Yq11.222	0.099	6.00E-06
208067_x_at	UTY	7404	Yq11	0.096	1.00E-05
230760_at	ZFY	7544	Yp11.3	0.093	1.60E-05
*204410_at*	*EIF1AY*	9086	Yq11.223	0.093	1.80E-05
*224589_at*	*XIST*	7503	Xq13.2	0.092	2.90E-05
205048_s_at	PSPH	5723	7p11.2	0.090	4.00E-05
214131_at	TXLNG2P	246126	Yq11.222	0.089	4.70E-05
207805_s_at	PSMD9	5715	12q24.31	0.089	5.60E-05
238900_at	HLA-DRB1	3123	6p21.3	0.088	6.70E-05
1559003_a_at	CCDC163P	126661	1p34.1	0.088	7.10E-05
208909_at	UQCRFS1	7386	19q12	0.086	0.000112
215333_x_at	GSTM1	2944	1p13.3	0.085	0.000158
208919_s_at	NADK	65220	1p36.33	0.085	0.000163
241808_at	ZC2HC1A	51101	8q21.12	0.082	0.000287
225318_at	---	---	---	0.081	0.000345
212262_at	QKI	9444	6q26	0.081	0.000379
225236_at	RBM18	92400	9q33.2	0.081	0.000434
206279_at	PRKY	5616	Yp11.2	0.080	0.000554
1554094_at	ENTPD5	957	14q24	0.080	0.000554
203280_at	SAFB2	9667	19p13.3	0.080	0.000574
226990_at	CAPRIN1	4076	11p13	0.079	0.000628
203056_s_at	PRDM2	7799	1p36.21	0.079	0.000726
241033_at	---	---	---	0.078	0.000844
205173_x_at	CD58	965	1p13	0.078	0.000872
235104_at	ERAP2	64167	5q15	0.078	0.000882

The table shows probe sets with an empirical p-value < 0.001, sorted by descending dip test statistics. About 47% of the genes in the list are located on heterosomes, among those all genes of the gender marker “REDKX” ([6], italicized for visualization purposes). Probe sets for HLA-genes, which were further considered during the marker validation process, are highlighted in bold.

**Table 2:** Allele-specificity of HLA-DQA1 probe sets

**Gene**	**HLA-DQA1**																					
**Probe set**	**203290_at**											**213831_at**										
**Probe**	**1**	**2**	**3**	**4**	**5**	**6**	**7**	**8**	**9**	**10**	**11**	**1**	**2**	**3**	**4**	**5**	**6**	**7**	**8**	**9**	**10**	**11**
Allele (predicted)																						
HLA-DQA1*0101.1	0	0	0	0	0	***1***	0	0	0	0	0	***1***	***1***	***1***	***1***	***1***	0	0	***1***	***1***	***1***	***1***
HLA-DQA1*0102	0	0	0	0	0	***1***	0	0	0	0	0	***1***	***1***	***1***	***1***	***1***	***1***	***1***	0	0	0	***1***
HLA-DQA1*0103	0	0	0	0	0	***1***	0	0	0	0	0	***1***	***1***	***1***	***1***	***1***	***1***	***1***	***1***	***1***	***1***	***1***
HLA-DQA1*0201	0	0	0	0	0	0	0	0	0	0	0	***1***	0	0	0	0	0	0	0	0	0	0
HLA-DQA1*0301.1	0	0	0	0	***1***	0	0	0	0	0	0	***1***	0	0	0	0	0	0	0	0	0	0
HLA-DQA1*0401	***1***	***1***	***1***	***1***	***1***	***1***	***1***	***1***	***1***	***1***	***1***	0	0	0	0	0	0	0	0	0	0	0
HLA-DQA1*0501	***1***	***1***	***1***	0	0	***1***	***1***	***1***	***1***	***1***	***1***	0	0	0	0	0	0	0	0	0	0	0

The nucleotide sequence of 203290_at perfectly matches the *0401 allele, and contains at least one mismatched probe for other alleles while 213831_at perfectly matches the *0103 allele, and contains at least one mismatched probe for other alleles. Perfect matches are indicated as an italicized bold 1. Allele sequences from [12].

**Table 3:** Application of the score to public datasets

**Individual**	**Sample description**	**CEL file ID**	203290_at HLA-DQA1 *0401	213831_at HLA-DQA1 *0103	**209728_at HLA-DRB4**	**HLA-Score**	**REDKX gender QC**	**Flag**	**Reason for flag**	**Decision**
55	Time point 1	GSM155503.CEL	28	4182	48	010	F			
	Time point 2	GSM155504.CEL	23	4606	72	010	F			
	Time point 3	GSM155505.CEL	10	2412	24	010	F			
	Time point 4	GSM155506.CEL	28	4765	21	010	F			
54	Time point 1	GSM155499.CEL	33	516	985	011	F			
	Time point 2	GSM155500.CEL	49	681	1245	011	F			
	Time point 3	GSM155501.CEL	28	1073	3142	011	F			
	Time point 4	GSM155502.CEL	26	914	2573	011	F			
45	Time point 1	GSM155495.CEL	45	8041	187	**011**	F	HLA-score	Intensity of 209728_at HLA-DRB4 slightly above threshold	Not critical
	Time point 2	GSM155496.CEL	59	7619	123	010	F			
	Time point 3	GSM155497.CEL	32	6385	105	010	F			
	Time point 4	GSM155498.CEL	59	7062	40	010	F			
35	Time point 1	GSM155475.CEL	1218	42	6444	**101**	**F**	HLA-score and REDKX QC-score	Gender different for patient samples	Possible sample mix-up; follow up
	Time point 2	GSM155476.CEL	436	1491	231	111	M			
	Time point 3	GSM155477.CEL	508	1751	200	111	M			
	Time point 4	GSM155478.CEL	126	113	5	**000**	M	HLA-score	Intensity of 209728_at HLA-DRB4 and 213831_at HLA-DQA1 10× to 46× smaller than those from other patient samples	Possible sample mix-up; follow up
32	Time point 1	GSM155471.CEL	1235	32	8231	101	F	HLA-score and REDKX QC-score		
	Time point 2	GSM155472.CEL	420	1878	291	**111**	**M**		Gender different for patient samples	Possible sample mix-up; follow up
	Time point 3	GSM155473.CEL	1807	49	7332	101	F			
	Time point 4	GSM155474.CEL	1128	42	7122	101	F			

Each sample of the dataset is labelled with a three-digit score (one “1” or “0” flag per probe set). The presence of intra-score differences elicits further follow-up investigation as to the nature and source of the difference. Intensity thresholds mark an “on” or “off”-status of the transcript expression, flagged as “1” or “0”, respectively. The thresholds were empirically determined for each marker separately. In some instances, the score difference is due to mild threshold violation, in other instances it may be due to sample mix-ups. The score picks up those samples which are being flagged by the REDKX gender QC, and detects further samples with issues (labeled in bold). REDKX panel expression values are provided in Additional file 1: Table S1.

Table 4 summarizes the findings in 188 samples from 53 donors in five studies of four tissues types, whole blood, peripheral blood mononuclear cells (PBMC), and lung biopsy. Six samples were flagged for suspicious gender- and/or donor ID annotation, five of which in a single dataset. Three samples had incorrect gender annotation according the REDKX gender QC. Based on the HLA-score results, seven samples had potentially incorrect donor identifiers. The annotation of 5 of the six flagged samples could not be resolved given the information resources provided in the public domain. Three of those five samples had incorrect gender and donor ID annotation, two presented HLA-score data which could not be explained without further investigation, which was beyond the scope of the current project. In summary, based on the HLA-score and the REDKX gender QC, 3% of the samples are not usable for further analysis and would yield incorrect results.

**Table 4:** Summary of the score tests

	**Dataset ID**	**Tissue**	**Number of samples**	**Number of donors with repeat measures**	**Flagged samples**	**Reason for suspicion**		**Fail after inspection (not resolvable)**	**Decision**
						**REDKX- gender QC fail**	**Score differences (intra-donor)**		
						**N° samples**	**N° samples**	**N° samples**	
	GSE6751	PBMC	59	15	5	3	5	4	Follow-up
	GSE6281	Skin	33	11	0	0	0	0	NA
	GSE20489	Whole blood	54	11	1	0	1	1	Follow-up
	GSE24206	Lung	12	6	0	0	0	0	NA
	GSE32473	Skin	30	10	0	0	0	0	NA
Summary	5 datasets	4 tissues	188	53	6	3	6	5	

The candidate markers were applied to datasets from the public domain (http://www.ncbi.nlm.nih.gov/geo). About 3% of samples have annotation issues which could not be resolved by visual inspection of the intensity data alone. The individual REDKX marker intensity values are not shown. Individual results are shown in Additional file 1: Table S1.

## Discussion

Notwithstanding technical precision, annotation precision is a major component contributing to high data quality. In an effort to standardize annotation, metadata databases have been developed which provide user guidance by implementation of controlled vocabulary [13]. However, Quantile-quantile plot of Hartigans’ dip test statisticshuman error and subjectivity is still a common source of incorrectness in such databases [1]. Analysis errors and wrong conclusions with possibly detrimental consequences can be the result if annotation errors remain undetected [4]. Current strategies of dealing with annotation errors include statistical tests at a relatively late stage of the analysis. We hypothesized that transcript markers could aid in improving detection of annotation precision at an early stage, at best before the analysis.

Our approach was to investigate, design, develop and implement quality control tools to qualify microarray data in the context of the donor sample. As an initial step we have recently developed and qualified the so-called REDKX marker which is a transcript panel marker based on expression of genes located on heterosomes, indicating gender of sample donors in clinical microarray studies [6]. However, in studies with multiple samples from donors, correct gender annotation still leaves an uncertainty about the correct assignment of a sample to a donor, as samples with the same gender annotation may come from different subjects of that gender. Hence, we devised a second annotation transcript quality control marker, which would increase the detectability of samples with donor identification mislabels.

By applying the Hartigans’ Dip Test statistic we identified probe sets with bimodal expression pattern. Of particular interest are probe sets which show allele-selectivity, such as probe sets for histocompatibility genes HLA-DQA1 and HLA-DRB4. Both genes are located on the p21 arm of chromosome 6, and could be in a linkage disequilibrium region. The markers do not represent expression quantitative trait locus (eQTL) genes, and are expressed independent of gender. HLA genes code for cell surface proteins which are expressed by antigen presenting cells and in the immune system serve the purpose of self- vs. nonself-discrimination [14]. Hence, HLA gene expression patterns represent highly specific “fingerprinting” of individual donors. In general, our results receive support in the independent findings of Joehanes et al. who have suggested that gene expression levels, including those of HLA genes, could serve as “fingerprinting” data in microarray datasets [15]. The authors came to this conclusion as part of their assessment of gene expression analysis from different blood-derived RNA sources. Here, we identify a candidate marker consisting of HLA genes in an unsupervised analysis and qualify it by training and testing its performance on a number of publically available datasets.

HLA genes are essential contributors to susceptibility to risk or resistance to several autoimmune diseases such as multiple sclerosis, rheumatoid arthritis, and Type 1 Diabetes [16-18]. As sequencing of the HLA locus continues and more alleles are being identified, the number of allele-disease associations will grow [19-22]. For our exploratory marker analysis we applied expression data for three HLA probe sets. As a consequence of the extremely high multiplexing of parameters interrogated by a microarray it is not surprising that the behaviour of a single transcript in a large transcript population is not always 100% predictable and is the result of multiple factors such as assay and array performance. A three-digit sample identifier score yields 8 different score types, not sufficient to completely discriminate all donors in studies with 9 or more donors. However, as shown in the case of donor 32 in GSE67511, only the combination of the REDKX gender marker and the score has the potential to further reduce sample ID ambiguities by eliciting follow-up investigations should discrepancies between scores within a sample collection of a single donor arise. In the present analysis, we found that about 5% of the publically available samples used herein rewarded follow-up investigations, where half of those cases could not be resolved by interrogating the data alone. Despite our encouraging results from public datasets of 4 tissue types, we recommend the optimal intensity thresholds be developed related to each tissue of interest. The reason for this suggestion lies in the tissue dependent expression level variations as well as in technical dissimilarities, which may lead to deviations in global signal intensity, e.g. by different scaling settings.

Context-dependent biomarker qualification is driven by the application of the marker. The proposed genomic fit-for purpose biomarker approach for quality control of sample ID annotations in transcriptomics clinical datasets with multiple samples per donor could be applied immediately. As part of our approach to this end, we have implemented the haplotype-based quality control as part of our microarray quality control pipeline. Microarray data files are automatically reviewed by the software after they are produced, with the analysis results cached and made available through a web interface, where likely problems are highlighted for further investigation.

In case of detected mismatches to reported metadata, a root-cause analysis will be necessary to determine the reason for the error. If the cause cannot be determined in too many cases or is systematic, the decision could be not to use the entire dataset [1].

In conclusion, we have identified a set of transcripts, which, particularly in combination with the REDKX gender marker, is capable that can be used as a starting point to control for sample ID annotation errors in clinical datasets with multiple samples per donor. In publically available datasets we have identified about 3% of unresolvable annotation errors. Thus we recommend applying the marker broadly in transcriptomics studies and to follow-up with root cause analysis where necessary. Direct sequencing will provide further confirmation and possible expansion of the marker panel.

## Conclusions

Hartigans’ Dip Test statistic is able to robustly identify probe sets with non-unimodal expression patterns, namely 203290_at (HLA-DQA1*0401), 213831_at (HLA-DQA1*0103), and 209728_at (HLA-DRB4). Combination of the so-called HLA-score and the REDKX marker panels provides a useful molecular quality control metric for sample annotation in clinical transcriptomics studies. Biological or clinical interpretation of gene expression data should take the haplotype specificity of these probe sets into account.

## Methods

### Data analysis

Publically available microarray datasets were downloaded from the data repository Gene Expression Omnibus (GEO, http://www.ncbi.nlm.nih.gov/geo/). Data analysis was performed in R [23]. The training of the marker was performed on MAS5 normalized data. The trimmed mean intensity (2%) per HG-U133 plus 2 array (http://www.Affymetrix.com, http://www.affymetrix.com/catalog/131455/AFFY/Human-Genome-U133-Plus-2.0-Array#1_1, last accessed 03/2014) was scaled to 150. Intensity based filtering was applied using the criteria that the 90th percentile had to be greater than 6 on log2 scale. The dip test statistics were calculated for every probe set of 47 Affymetrix HG-U133_Plus_2 microarrays from a study of systemic juvenile idiopathic arthritis (GSE7753, http://www.ncbi.nlm.nih.gov/geo) using the R package dip test (version 0.75-5, http://cran.r-project.org). To compute empirical p-value assessing the significance of an individual dip test statistic value, the dip test statistic was computed with 1 × 10^6^ permutations per probe set on a simulated dataset of the same sample size as the microarray training dataset. A proportion of simulated dip test statistic values that were greater than the observed one was used as an empirical p-value. Probe sets with empirical p-values < 0.001 were further considered. Empirical p-values were adjusted using the false discovery criterion by Benjamini and Hochberg [24]. For each probe set the interquartile range (IQR, 95th to 5th quantile) was calculated. Dip test core cut-offs using the training set data intensity-thresholds for each individual marker were empirically determined based on the distributions of the mean-trimmed MAS5 normalized expression (see above).

## Competing interests

The authors declare that they have no competing interests.

## Authors’ contributions

NH conceived of the study design and data generation; EL performed data analysis and interpretation of the data; EK performed the marker discovery and data analysis; JS conceived of the study and the design of the QC tool; LB generated microarray data; JP was involved in study design and design of the QC tool; AS performed data analysis and manuscript writing; NRN gave final approval of the manuscript; FS revised the manuscript critically for important intellectual content and gave approval of the manuscript. All authors read and approved the final manuscript.

## Supplementary Material

Additional file 1: Table S1 Normalized signal intensities for marker probe sets and scores. For 188 test samples, normalized signal intensities of the marker probe sets of the HLA-score and the REDKX marker are shown, along with the panel scores. Click here for file
